# The Supratrochlear Artery: Anatomy, Variations, and Clinical Relevance in Aesthetic Surgery

**DOI:** 10.7759/cureus.92563

**Published:** 2025-09-17

**Authors:** Xheni Merizaj, Niko Haxhiu, George Tsakotos, Dimosthenis Chrysikos, Theodore Troupis

**Affiliations:** 1 Thoracic Surgery, KAT Attica General Hospital, Athens, GRC; 2 1st Orthopaedics Department, KAT Attica General Hospital, Athens, GRC; 3 Anatomy, National and Kapodistrian University of Athens School of Medicine, Athens, GRC

**Keywords:** aesthetic facial surgery, anatomical structure, anatomical variability, forehead flaps, supratrochlear artery, supratrochlear foramen

## Abstract

The supratrochlear artery (STA) is one of the terminal branches of the ophthalmic artery, coursing through the medial forehead and glabellar region to supply the central upper face. Although relatively small in caliber, it holds considerable clinical importance because of its anatomical variations and its communication with the ophthalmic circulation. These features make the STA especially relevant in both reconstructive procedures and aesthetic interventions.

In this review, we examined the anatomy of the STA with a focus on its origin, branching characteristics, vessel diameter, depth variations, and anastomotic networks. We also explored its role in reconstructive surgery and aesthetic medicine. A systematic search was performed in the PubMed online database.

Considerable variability was noted in its course and branching, including clinically significant intracranial-extracranial anastomoses with the angular, supraorbital, and dorsal nasal arteries. The literature included evidence from cadaveric dissections, Doppler ultrasonography, and angiographic studies, each of which contributed to a clearer picture of the vessel’s patterns.

From a surgical standpoint, case series emphasized the STA’s central role in surgical reconstructions. On the other hand, aesthetic practice has highlighted the risks associated with its inadvertent cannulation during soft-tissue filler injections. Accidental intravascular injection into the STA or its anastomotic network has been linked to devastating outcomes, including vision loss.

Overall, a detailed understanding of the STA’s anatomy is essential for safe and effective practice. The combination of classical dissection data with modern imaging has improved preoperative planning and may reduce complications in minimally invasive procedures. This review brings together current knowledge on the STA, emphasizing its dual identity as both a valuable surgical vessel and a high-risk structure in aesthetic medicine.

## Introduction and background

The supratrochlear artery (STA) holds a key position in the vascular anatomy of the upper face and forehead. It is one of the two terminal branches of the ophthalmic artery, itself a direct branch of the internal carotid system [1]. Originating near the trochlea, the STA ascends almost vertically, passing through the corrugator and frontalis muscles before supplying the medial forehead, the glabellar region, and the surrounding soft tissues [2-4]. Although relatively small in caliber, this vessel has long been recognized as clinically significant. Its size belies its importance, as it is indispensable in forehead-based reconstructive flaps and, at the same time, represents one of the most vulnerable arteries during aesthetic procedures. Because its diameter, depth, course, and branching can vary considerably between individuals, a detailed understanding of these features is essential in both surgical and minimally invasive practice [5].

From a developmental standpoint, the STA emerges early in craniofacial vascular formation. The ophthalmic artery, from which the STA derives, begins to take shape by the fourth week of gestation [6]. During this period, orbital vascularization is supplied by a primitive plexus, including the dorsal and ventral ophthalmic arteries, both initially in continuity with the internal carotid circulation. As the embryo develops, the dorsal ophthalmic artery gradually becomes dominant and gives rise to the definitive ophthalmic artery, which later branches into the STA [7]. By approximately the eighth week of gestation, the STA is identifiable as a distinct vessel projecting forward into the developing forehead and glabella [8-10]. At the same time, its superficial and deep branches differentiate alongside the maturing soft tissues: the superficial component integrates into the subcutaneous layers, while the deep branch aligns with the frontalis muscle and periosteum of the frontal bone [11]. This dual organization ensures blood supply to both skin and musculature of the upper face. Small deviations in vascular remodeling at this stage can explain the diversity of STA patterns observed in adulthood, ranging from variations in branching to alternative depths and points of origin [5]. The parallel development of the STA and supraorbital artery, which share embryonic pathways and form early anastomoses, further accounts for the complex interconnections often encountered in the forehead region [8, 9].

The clinical relevance of the STA has been documented for more than a century. Keller, in the nineteenth century, first highlighted its contribution to forehead and scalp circulation [6]. Subsequent cadaveric dissections, notably those of Kleintjes and Cordova et al., refined the description of its course, branches, and anastomotic patterns [7, 11]. These studies consistently emphasized the presence of extensive intracranial-extracranial communications, particularly with the angular, supraorbital, and dorsal nasal arteries. Such connections, while valuable for collateral circulation, also represent potential routes for embolic events in aesthetic practice. The artery’s importance in reconstructive surgery became firmly established with the introduction of the paramedian forehead flap by Shumrick, a technique that has since undergone numerous modifications for nasal and periorbital reconstruction [3, 12]. A precise appreciation of the STA’s depth, diameter, and trajectory has proved essential in optimizing flap viability and reducing the risk of ischemic complications [13].

The increasing popularity of non-surgical facial rejuvenation has made knowledge of the STA even more critical [6, 7, 14, 15]. Its relatively superficial course in the glabellar and medial forehead regions places it at high risk during filler injections [15-18]. The danger is compounded by its anastomoses with the ophthalmic and retinal arteries, which provide a direct pathway for retrograde embolization. Such events have been implicated in some of the most devastating complications in aesthetic medicine, including irreversible vision loss, stroke, and cutaneous necrosis [3, 5]. The widespread use of hyaluronic acid fillers in the glabellar and forehead regions has therefore driven renewed anatomical and imaging investigations into the artery [14, 15]. Cadaveric studies, Doppler ultrasonography, and three-dimensional angiographic analyses have all been employed to clarify its size, depth, and branching variations across populations [7, 11].

Despite the volume of research, inconsistencies remain evident. Reported findings vary between cadaveric and imaging-based studies, and inter-population differences complicate the picture further. Methodological heterogeneity across studies also makes it difficult to reach definitive conclusions. Recent systematic reviews have highlighted the need for a consolidated synthesis of the available evidence [5, 19]. Such integration is especially relevant given the artery’s dual role: as a dependable pedicle for reconstructive flaps and, conversely, as a high-risk vessel in aesthetic medicine [5].

For these reasons, the present review sets out to provide a comprehensive synthesis of current knowledge regarding the STA. It will address its common variations and topographical relationships while also considering its clinical implications in both reconstructive and aesthetic surgery. By drawing together data from cadaveric dissections, angiographic imaging, Doppler ultrasonography, and clinical outcome reports, this review aims to equip surgeons, dermatologists, and aesthetic practitioners with a consolidated reference to support safer and more effective practice.

## Review

Study design

A systematic search was performed across PubMed up to June 2025. Search terms included combinations of “supratrochlear artery”, “anatomy”, “variations”, and “supratrochlear artery flaps”. From 145 original articles, a total of 41 were included in this review. Inclusion criteria: cadaveric anatomical dissections, imaging studies (angiography, CTA, Doppler ultrasound), and clinical studies involving STA-based flaps or reporting filler-related complications. Exclusion criteria: non-human studies, case reports unrelated to STA anatomy, and articles without primary data.

**Figure 1:**
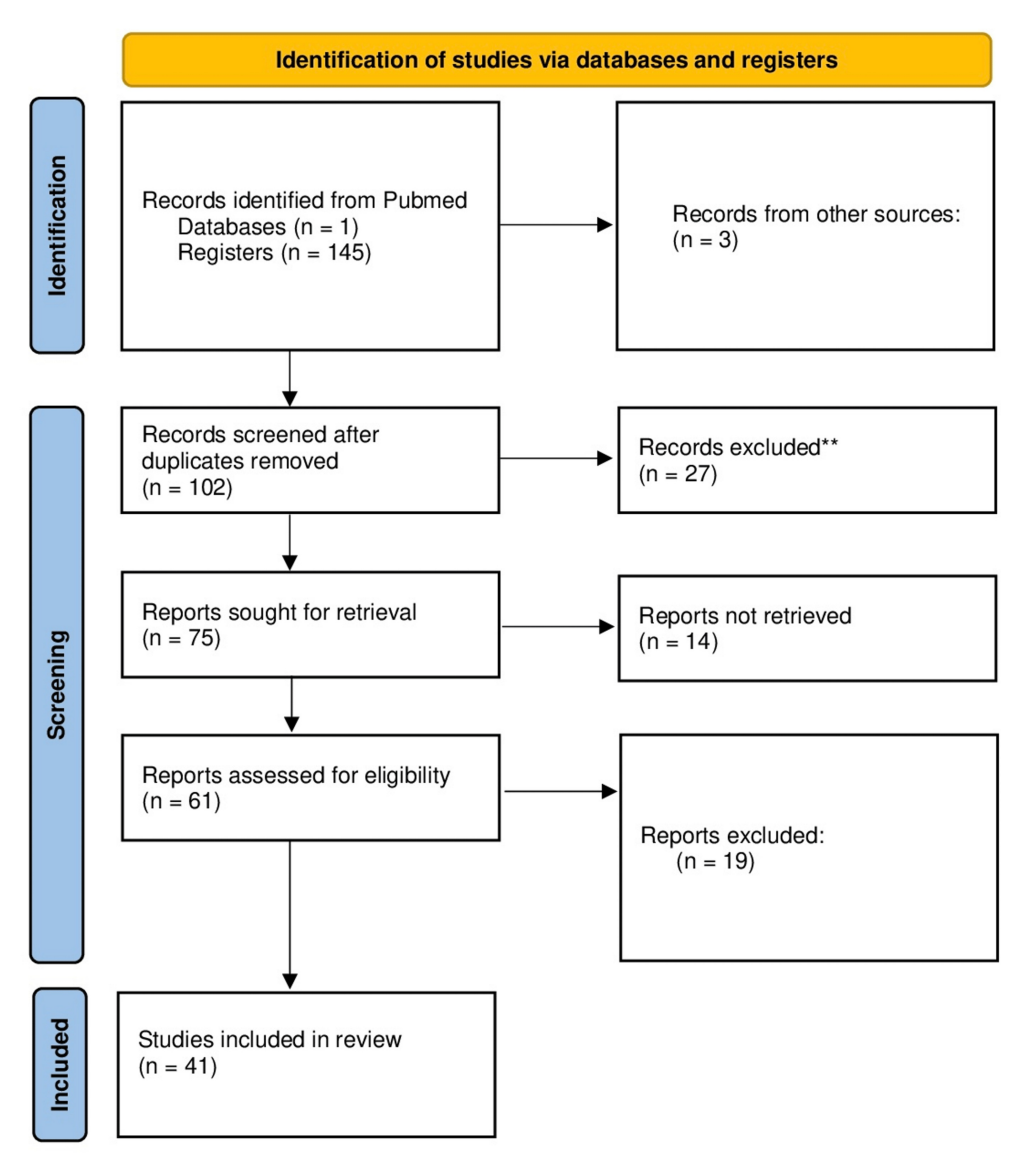
2020 flow diagram

Two independent reviewers extracted: sample size, hemifaces, demographics, STA origin, diameter, branching, depth, anastomoses, and clinical implications. The process is demonstrated in Figure 1 (2020 Prisma flowchart).

Origin

Cadaveric and imaging-based studies have provided complementary insights into the origin, caliber, and exit point of the STA. Schwenn and colleagues, through cadaveric dissections with sonographic correlation, confirmed by retrograde cannulation that both the STA and supraorbital artery communicate directly with the ophthalmic artery at the orbital apex, highlighting the potential for retrograde embolization [20]. In their series, the STA measured an average external diameter of just over 1.0 mm, slightly larger than the supraorbital artery, and typically emerged about 16 mm from the midline. Zhao et al., using three-dimensional CT angiography, demonstrated a degree of variability in arterial origin, with most STAs arising from the ophthalmic artery itself, a smaller proportion from the trochlear branch, and occasional origins from the supraorbital or angular arteries [14]. These findings broadly align with earlier cadaveric reports that described the ophthalmic artery as the dominant source in the overwhelming majority of cases, with rare contributions from the angular or supraorbital arteries. Morphometric analysis by Edizer et al., in turn, corroborated a mean STA caliber close to 0.9-1.0 mm, with the supraorbital artery averaging around 1.0 mm and the dorsal nasal artery approximately 0.8 mm [4]. Their dissections also underscored the redundancy of the periocular arterial network, with the STA contributing to anastomotic arcades that often involve the superficial temporal artery. Regional anatomical studies have further detailed exit patterns, noting that the STA and supraorbital artery most frequently emerge through separate foramina or notches, an arrangement present in the majority of foreheads examined, and enter the frontalis muscle shortly thereafter. When considered together, these series converge on a consistent anatomical picture: the STA is a vessel of approximately 1.0-1.2 mm in caliber at the orbital rim, typically exiting 15-18 mm from the facial midline, and displaying modest but clinically important variation in its origin [3, 4, 21-23].

Depth and course

In Doppler ultrasonography across the glabellar frown lines, Cotofana et al. quantified depth gradients and course variability of the STA in vivo, providing the kind of point-by-point mapping now favored for filler safety. Their dataset reports typical STA depths ranging on the order of ~2-6 mm depending on the exact glabellar location (with site-specific means tabulated), and the proportion of hemifaces with relatively superficial (<3 mm) vs deeper (>6 mm) positions-parameters that meaningfully change injection strategy [17]. In another Doppler series of 74 hemifaces, Shen et al. measured inner diameters 0.6-1.0 mm and peak systolic velocities 9.2-24.9 cm/s for the STA/supraorbital(SOA)/dorsal nasal/angular arteries, with high detection rates across periorbital segments-again illustrating the live variability clinicians face [17, 23].

**Figure 2:**
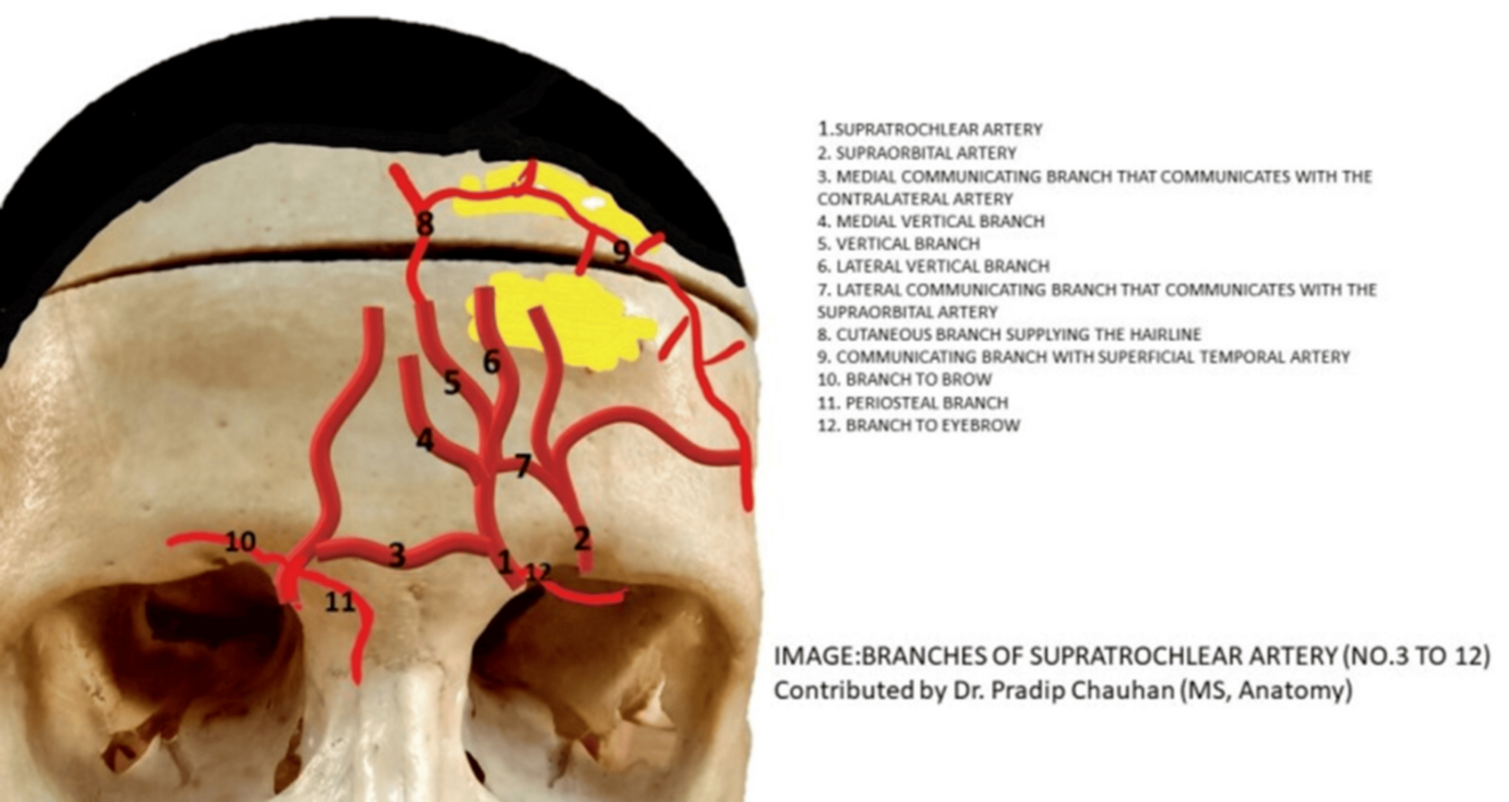
Supratrochlear artery branches Contributed by P Chauhan, MD: From: Anatomy, Head and Neck, Supratrochlear Copyright © 2025, StatPearls Publishing LLC. Free distribution. https://www.ncbi.nlm.nih.gov/books/NBK557549/figure/article-29753.image.f4/

The STA demonstrated variable branching patterns across the included studies, with most authors classifying them into two to four distinct subtypes [11, 14, 16, 22-27]. In cadaveric dissections, a simple two-subtype model was initially proposed, distinguishing a single undivided trunk (38-41%) from a bifurcated configuration (59-62%) [5, 28]. More detailed analyses, incorporating both dissection and computed tomographic angiography, identified a three-subtype system, comprising a single trunk (33-40%), bifurcation into superficial and deep branches (48-54%), and trifurcation or multiple small twigs (12-14%) [29-31]. High-resolution three-dimensional CT studies further refined this classification into a four-subtype model, adding a rare Type IV variant characterized by early division at the orbital rim or formation of an arcade with the supraorbital artery, observed in less than 5% of cases [26]. When data were pooled, the most frequent configuration was bifurcation (~50-55%), followed by a single trunk (~35-40%), while trifurcation (~10-15%) and arcade-type variants (<5%) were uncommon. The following picture shows the named branches of the STA as known in the international bibliography (Figure 2).

Surface Landmarks and the Supratrochlear Pedicle

Vural et al. compared glabellar frown lines (GFLs) with the supratrochlear vascular pedicle (SVP) in 19 volunteers (Doppler) and 8 cadaver heads. The SVP lay at the GFLs in ~50%, and was on average 3.2 mm lateral to the GFLs in the remainder - identifying a reliable surface landmark for planning paramedian forehead flaps when Doppler is equivocal. Complementing this, Ugur et al. demonstrated that the medial canthus represents a consistent external landmark for localizing the STA. In their study, the STA typically emerges just superior and slightly lateral to the medial canthus before ascending beneath the frontalis. This relationship allows surgeons to anticipate the artery’s course during flap elevation, minimize inadvertent vascular injury, and plan safe injection zones or flap pedicles with greater precision. Using the medial canthus as a reference is particularly valuable when soft-tissue landmarks like the GFLs are obscured by edema, dressings, or previous surgery [31, 32].

Anastomoses and Functional Connectivity

In a functional anastomosis study, Kelly et al. combined latex injections and flap elevation in nine fresh-frozen heads, demonstrating consistent STA anastomoses with the facial/angular system and providing a mechanistic rationale for low-loss rates in forehead flaps pedicled below or at the medial canthus. The work empirically supports a cross-territorial inflow that maintains perfusion even when the proximal pedicle is narrowed, and highlights routes for potential retrograde embolization from superficial facial vessels into the ophthalmic territory [33, 34].

Cadaveric anatomic dissections and imaging consistently corroborate extensive anastomotic links among STA, SOA, angular/dorsal nasal, and superficial temporal branches, consistent with Edizer et al. and Zhao et al. noted above [4, 14]. These connections are a double-edged sword: they underpin flap reliability, but also provide multiple reflux pathways during high-pressure filler injections.

Reconstructive Reliability

Multiple series document forehead-based nasal reconstruction with high survival [35]. A preliminary experience with full-thickness, bivalved paramedian forehead flaps in five patients reported 100% flap survival, with four minor complications resolving conservatively, illustrating the feasibility and robustness of large/complex defects. Broader reconstructive practice has long leveraged STA-based pedicles as the workhorse for major nasal defects [33, 36].

STA-based island flaps have expanded indications beyond classic two-stage techniques. A recent comparative clinical study of periorbital defect reconstruction by Fang et al. evaluated 68 patients, comparing temporal, forehead (STA/SOA), and buccal island flaps. The authors reported detailed rates of complications (e.g., partial distal congestion/necrosis) and functional/aesthetic outcomes across flap categories (with per-group n values and percentages tabulated), concluding that forehead (STA/SOA) flaps are practicable and manipulable with favorable outcomes for appropriate defect patterns [37-39].

Filler-Related Ophthalmic Events and Mitigation

Aesthetically, the STA is frequently implicated in ophthalmic artery embolism. A clinical analysis by Cong et al. collated 20 cases of filler-related ophthalmic events, detailing injection sites, agents, measured periorbital arterial distances (e.g., 4.1-5.1 mm depths cited for high-risk planes in the glabella/medial forehead), and outcomes. [13]. The pattern of sudden vision loss with immediate pain underscores a pressurized retrograde mechanism through STA/SOA branches into the ophthalmic and central retinal circulation. Global literature reviews describe similar patterns of injury and permanent monocular blindness after glabellar and nasal root injections, consolidating the STA’s role in the highest-risk aesthetic zones [13, 17, 26, 40-42].

Technique refinements increasingly emphasize live vessel mapping and flow-limiting maneuvers. Mespreuve et al. used cross-sectional angiographic imaging to visualize variable facial arterial anatomy relevant to filler safety and even proposed augmented-reality guidance for high-risk corridors-practicality aside, this supports the concept of individualized vascular maps over “average anatomy” [43]. In parallel, Cotofana et al. and Shen et al. quantify in vivo depth and diameter/velocity parameters at injector-relevant points, translating cadaveric anatomy into real-time sonographic guidance [17, 24].

Finally, the concept of periorbital digital compression has re-emerged. Hwang and Han analyzed plastinated specimens and the anatomical literature to localize the origin of the nasal branch of the ophthalmic artery and argued for precise digital compression during high-risk injections to reduce reflux - a pragmatic adjunct to, not a replacement for, ultrasound guidance and low-pressure technique [44].

Discussion

A Unifying Model of STA Risk and Reliability

The same features that make the STA a superb reconstructive pedicle, caliber consistency ~1.0-1.2 mm at the rim, predictable exit ~15-18 mm from midline, robust anastomoses, are precisely those that render the glabella and medial forehead the most dangerous filler zones. Schwenn et al. demonstrated the anatomical plausibility of retrograde embolization experimentally with ink tracking from STA/SOA to the ophthalmic artery; their exact numbers (STA 1.08 ± 0.19 mm, exit 16.4 ± 1.7 mm) now exist as anchors for surgical planning and safety counseling [20]. Zhao et al. add that multiple STA origins (65% trochlear, 30% direct, 5% via SOA) increase route redundancy, not only for perfusion but for reflux during inadvertent intravascular injections [14].

Layer-by-Layer: Depth and Plane Selection

Doppler work moves the field beyond static cadaveric averages toward patient-specific depth and hemodynamics. Their measurements confirm that the STA’s depth is not fixed: at some glabellar points it lies as shallow as ~2-3 mm, while at others, deeper ~5-6 mm planes predominate, differences large enough to flip an otherwise “safe” injection superficial to intravascular if technique is not adjusted to the site [17, 24]. This is reinforced by Phumyoo et al., who provide specific horizontal offsets (14.7 and 19.2 mm) and depths (4.2 and 5.9 mm) for the STA’s superficial/deep branches at the glabella, which is directly translatable to ultrasound-guided procedural planning [18].

Landmarks and Doppler Synergy for Flaps

For reconstructive surgeons, Vural’s observation that the SVP lies at the GFLs in ~50% and ~3.2 mm lateral otherwise provides simple surface guidance that complements Doppler when acoustic windows are poor (e.g., edema, dressings) [31]. Intraoperative reassurance about functional anastomoses supports the safety margin of paramedian forehead flaps even when the pedicle must be narrowed or routed around scarred tissue [30, 32-33]. These insights integrate well with modern island-flap variants, where Fang et al.’s outcomes across 68 patients demonstrate that forehead (STA/SOA) island flaps remain high-yield options with manageable complication profiles for periocular defects [38].

Why Ophthalmic Events Persist - and How to Reduce Them

Despite improved awareness, ophthalmic artery embolism continues to occur. Even experienced injectors can precipitate retrograde emboli with small boluses and ordinary hyaluronic acid syringes when injecting near the glabella/root. Their collated depths (≈4-6 mm) at high-risk points mirror Phumyoo and Cotofana/Shen measurements, underscoring that misjudging the plane by millimeters can determine whether filler tracks subcutaneously or intravascularly. Reviews further document the persistence of blindness cases, with the STA among the most commonly implicated routes in medial forehead and nasal root injections [15, 17, 18, 24].

A rational mitigation bundle, ultrasound mapping, blunt cannulas where appropriate, small aliquots, low pressure, constant needle motion, and well-placed digital compression (per Hwang & Han and prior expert commentary) has the greatest likelihood of reducing but not eliminating risk [44]. Finally, advanced imaging can serve high-risk or revision cases by exposing unusual variants (e.g., dominant trochlear origin, early bifurcation, atypical superficial course) beforehand.

## Conclusions

The STA is a vital structure in the vascularization of the upper face and forehead, with considerable clinical significance in aesthetic and reconstructive surgery. While its anatomical course is generally consistent, variations in origin, depth, and branching patterns underscore the need for individualized surgical and procedural planning. A thorough understanding of these anatomical details, combined with preoperative imaging, is essential to optimize patient outcomes and minimize complications.

## References

[1] (2016). Gray’s Anatomy, The Anatomical Basis of Clinical Practice.

[2] Koziej M, Polak J, Hołda J (2020). The arteries of the central forehead: Implications for facial plastic surgery. Aesth Surg J.

[3] (1989). Surgical anatomy of the orbit and periorbital region.

[4] Edizer M, Beden U, Icten N (2009). Morphological parameters of the periorbital arterial arcades and potential clinical significance based on anatomical identification. J Craniofac Surg.

[5] Agorgianitis L, Panagouli E, Tsakotos G, Tsoucalas G, Filippou D (2020). The supratrochlear artery revisited: An anatomic review in favor of modern cosmetic applications in the area. Cureus.

[6] Keller HM, Lüscher TF, Adorjani C, Vetter W (1984). Doppler analysis of blood flow of the supratrochlear artery: age specific pattern analysis in healthy and hypertensive patients. (Article in German). Ultraschall Med.

[7] Cordova A, D’Arpa S, Massimiliano T, Toia F, Moschella F (2014). A propeller flap for single-stage nose reconstruction in selected patients: Supratrochlear artery axial propeller flap. Facial Plastic Surg.

[8] Padget DH (1948). The development of the cranial arteries in the human embryo.

[9] Schonauer F, Taglialatela Scafati S, Molea G (2010). Supratrochlear artery based V-Y flap for partial eyebrow reconstruction. J Plast Reconstr Aesthet Surg.

[10] Safavi-Abbasi S, Komune N, Archer JB (2016). Surgical anatomy and utility of pedicled vascularized tissue flaps for multilayered repair of skull base defects. J Neurosurg.

[11] Kleintjes WG (2007). Forehead anatomy: Arterial variations and venous link of the midline forehead flap. J Plast Reconstr Aesthet Surg.

[12] Akdemir Aktaş H, Mine Ergun K, Tatar İ, Arat A, Mutlu Hayran K (2022). Investigation into the ophthalmic artery and its branches by superselective angiography. Interv Neuroradiol.

[13] Cong LY, Phothong W, Lee SH, Wanitphakdeedecha R, Koh I, Tansatit T, Kim HJ (2017). Topographic analysis of the supratrochlear artery and the supraorbital artery: Implication for improving the safety of forehead augmentation. Plast Reconstr Surg.

[14] Zhao WR, Wang HB, Luo CE, Kong XX, Zhan WF, Luo SK (2019). Three-dimensional computed tomographic study on the periorbital branches of the ophthalmic artery: Arterial variations and clinical relevance. Aesthet Surg J.

[15] Beleznay K, Carruthers JD, Humphrey S, Jones D (2015). Avoiding and treating blindness from fillers: A review of the world literature. Dermatol Surg.

[16] Tansatit T, Phumyoo T, Jitaree B (2018). Ultrasound evaluation of arterial anastomosis of the forehead. J Cosmet Dermatol.

[17] Cotofana S, Alfertshofer M, Frank K (2020). Relationship between vertical glabellar lines and the supratrochlear and supraorbital arteries. Aesth Surg J.

[18] Phumyoo T, Jiirasutat N, Jitaree B, Rungsawang C, Uruwan S, Tansatit T (2020). Anatomical and ultrasonography-based investigation to localize the arteries on the central forehead region during the glabellar augmentation procedure. Clin Anat.

[19] Kliniec K, Domagała Z, Kempisty B, Szepietowski JC (2024). Arterial vascularization of the forehead in aesthetic dermatology procedures: A review. J Clin Med.

[20] Schwenn OK, Wüstenberg EG, Konerding MA, Hattenbach LO (2005). Experimental percutaneous cannulation of the supraorbital arteries: Implication for future therapy. Invest Ophthalmol Visual Sci.

[21] Erdogmus S, Govsa F (2007). Anatomy of the supraorbital region and the evaluation of it for the reconstruction of facial defects. J Craniofac Surg.

[22] Trzeciak M, Gładysz T, Przybycień W (2025). The depth of arterial supply of forehead: A meta-analysis. Folia Morphol.

[23] Potparic Z, Fukuta K, Colen LB, Jackson IT, Carraway JH (1996). Galeo-pericranial flaps in the forehead: A study of blood supply and volumes. Br J Plastic Surg.

[24] Shen WW, Du JN, Ma JX, Xia YC, Cui LG (2023). Evaluation of supratrochlear, supraorbital and angular artery course variations and depth by Doppler ultrasound. Aesthetic Plast Surg.

[25] Cho KH, Dalla Pozza E, Toth G, Bassiri Gharb B, Zins JE (2019). Pathophysiology study of filler-induced blindness. Aesthet Surg J.

[26] Liao ZF, Cong LY, Hong WJ, Luo CE, Luo SK (2022). Three-dimensional computed tomographic study of the supratrochlear artery and supraorbital artery to determine arterial variations and their relationship. Dermatol Surg.

[27] Yoshioka N, Rhoton AL Jr (2005). Vascular anatomy of the anteriorly based pericranial flap. Neurosurgery.

[28] Fathi R, Biesman B, Cohen JL (2017). Commentary on: An anatomical analysis of the supratrochlear artery: Considerations in facial filler injections and preventing vision loss. Aesthet Surg J.

[29] Reece EM, Schaverien M, Rohrich RJ (2008). The paramedian forehead flap: A dynamic anatomical vascular study verifying safety and clinical implications. Plast Reconstr Surg.

[30] Skaria AM (2015). The median forehead flap reviewed: A histologic study on vascular anatomy. Eur Arch Otorhinolaryngol.

[31] Vural E, Batay F, Key JM (2000). Glabellar frown lines as a reliable landmark for the supratrochlear artery. Otolaryngol Head Neck Surg.

[32] Ugur MB, Savranlar A, Uzun L, Küçüker H, Cinar F (2008). A reliable surface landmark for localizing supratrochlear artery: Medial canthus. Otolaryngol Head Neck Surg.

[33] Park KH, Kim YK, Woo SJ (2014). Iatrogenic occlusion of the ophthalmic artery after cosmetic facial filler injections: A national survey by the Korean Retina Society. JAMA Ophthalmol.

[34] Kelly CP, Yavuzer R, Keskin M, Bradford M, Govila L, Jackson IT (2008). Functional anastomotic relationship between the supratrochlear and facial arteries: An anatomical study. Plast Reconstr Surg.

[35] Wang HY, Li QF, Gu B (2007). Anatomic study of supratrochlear artery and its application in nasal reconstruction. (Article in Chinese). Zhonghua Zheng Xing Wai Ke Za Zhi.

[36] Sedwick JD, Graham V, Tolan CJ, Sykes JM, Terkonda RP (2005). The full-thickness forehead flap for complex nasal defects: A preliminary study. Otolaryngol Head Neck Surg.

[37] Sharma RK (2011). Supratrochlear artery island paramedian forehead flap for reconstructing the exenterated patient. Orbit.

[38] Fang Z, Wu Y, Li J (2023). Feasibility, comparability and outcomes of three acquainted facial island flaps for periorbital defects reconstruction. Int Wound J.

[39] Fukuta K, Potparic Z, Sugihara T, Rachmiel A, Forté RA, Jackson IT (1994). A cadaver investigation of the blood supply of the galeal frontalis flap. Plast Reconstr Surg.

[40] Stigall LE, Bramlette TB, Zitelli JA, Brodland DG (2016). The paramidline forehead flap: A clinical and microanatomic study. Dermatol Surg.

[41] Khan TT, Colon-Acevedo B, Mettu P, DeLorenzi C, Woodward JA (2017). An anatomical analysis of the supratrochlear artery: Considerations in facial filler injections and preventing vision loss. Aesthet Surg J.

[42] Duman H, Sengezer M, Selmanpakoğlu AN, Eski M (2000). Supratrochlear artery flap for the repair of lower eyelid defects. Ann Plastic Surg.

[43] Mespreuve M, Waked K, Collard B, De Ranter J, Vanneste F, Hendrickx B (2021). The usefulness of magnetic resonance angiography to analyze the variable arterial facial anatomy in an effort to reduce filler-associated blindness: Anatomical study and visualization through an augmented reality application. Aesthet Surg J Open Forum.

[44] Hwang K, Han SH (2023). Digital compression of the origin of the nasal branch of the ophthalmic artery during filler augmentation: A review of the anatomical literature and an analysis of plastinated specimens. Journal of Craniofacial Surgery.

